# Authors’ Response to Peer Reviews of “Lessons Learned From the Resilience of Chinese Hospitals to the COVID-19 Pandemic: A Scoping Review”

**DOI:** 10.2196/36345

**Published:** 2022-04-06

**Authors:** Jack Stennett, Renyou Hou, Lola Traverson, Valéry Ridde, Kate Zinszer, Fanny Chabrol

**Affiliations:** 1 Centre Population et Développement Institute for Research on Sustainable Development IRD-Université de Paris Paris France; 2 Centre de recherche en santé publique Université de Montréal Montréal, QC Canada

**Keywords:** COVID-19, pandemic, SARS-CoV-2, health care, hospitals, health care strategy, hospital resilience, interventions, crisis response, crisis preparedness, public health

*This is the authors’ response to peer-review reports for “Lessons Learned From the Resilience of Chinese Hospitals to the COVID-19 Pandemic: Scoping Review”.*

## Round 1 Review

### Reviewer Anonymous [1]

#### General Comment

1. This paper’s [2] title mentions that the authors conducted a scoping review but used the PRISMA (Preferred Reporting Items for Systematic Reviews and Meta-Analyses) method. The authors should clarify the difference between scoping review and systematic review.
  Response: We have added a phrase in the text to explain the difference in more detail. References 14-16 explain the PRISMA scoping methodology.
2. Provide a table of the studies that were selected for final analysis (study title, publishing year, research methods, main findings of each study).
  Response: Done; this is contained in a .xls file.
3. State the study exclusion reasons clearly with a subheading in the Methods section.
  Response: We feel that the study exclusion criteria are implicit in the inclusion criteria.
4. Revise your study limitations according to the study inclusion and exclusion criteria
  Response: We have added two more sentences, which refer to the exclusion criteria.
5. Provide the list of all studies that were included at the initial stage without inclusion and exclusion limitations in a supplementary file.
  Response: We have a list of articles attached as an .xls file.
6. English editing is required.
  Response: Has been revised twice by native speakers.

### Reviewer AY [3]

1. The authors refer to Table 4 three times in the manuscript; however, this table is not attached to the document. The same occurs with Table 3 that is referred to on page 7, second paragraph.
  Response: These tables are within the supplementary files and are now labelled as such.
2. In relation to the PRISMA-ScR checklist, the eligibility criteria are not pointed out in the abstract section of the manuscript.
  Response: This has been done in the Inclusion Criteria section.
3. The registration number of the scoping review’s protocol is missing. The authors do not indicate if the protocol is available.
  Response: Please find the protocol here [4]; there is a link in the references.
4. There are some typos in the manuscript, for example:
  - Authors use the acronym PPE (personal protective equipment) several times in the manuscript. Please indicate its meaning the first time it appears in the manuscript (page 10).
  - In the abstract section, the word “found” appears in a smaller size than the rest of the words.
  Response: These issues have been resolved, and the manuscript was looked over again by a native speaker.

## Abbreviations

PPE: personal protective equipment
PRISMA: Preferred Reporting Items for Systematic Reviews and Meta-Analyses
PRISMA-ScR: Preferred Reporting Items for Systematic Reviews and Meta-Analyses Extension for Scoping Reviews
